# Coarse Aggregate Induced Fiber Dispersion and Its Role in UHPC Mechanics Across Flexural and Compressive Loading

**DOI:** 10.3390/ma18204796

**Published:** 2025-10-21

**Authors:** Chen Shen, Yue Zhang, Jianlin Li, Haonan Zeng, Changhui Yang, Linwen Yu

**Affiliations:** 1 National Engineering and Research Center for Mountainous Highways, Chongqing 400067, China; 2025995040@juwp.edu.cn (C.S.); lijianlin1@cmhk.com (J.L.); 15123604965@163.com (H.Z.); linwen.yu@cqu.edu.cn (L.Y.); 2 School of Civil and Architectural Engineering, Jiangxi University of Water Resources and Electric Power, Nanchang 330099, China; 3 College of Materials Science and Engineering, Chongqing University, Chongqing 400045, China; 4 State Key Laboratory of Bridge Safety and Resilience, China Merchants Chongqing Communications Research & Design Institute Co., Ltd., Chongqing 400067, China

**Keywords:** ultra-high-performance concrete, dispersion, flexural behavior, coarse aggregate, steel fiber

## Abstract

Ultra-high-performance concrete (UHPC) exhibits exceptional mechanical properties and durability but faces challenges such as high heat of hydration and limited stiffness. Incorporation of coarse aggregates offers a potential solution; however, it alters the dispersion of steel fibers, thereby affecting the mechanical performance of UHPC under different loading conditions. This study systematically investigates the influence of coarse aggregates on UHPC performance under different loading conditions, including four-point bending, uniaxial compression, and triaxial compression tests. The spatial distribution of steel fibers was quantitatively analyzed via image analysis to elucidate changes induced by CA incorporation. Results reveal that with 20 vol% coarse aggregate (10 mm), UHPC’s flexural strength is essentially unchanged (≈23 MPa), whereas flexural toughness decreases by about one-third. This toughness loss is linked to a slight increase in the fiber orientation angle (from 48.77° to 48.90°) and reduced continuity, which together weaken crack-bridging. Moreover, both flexural strength and toughness are governed primarily by the local steel-fiber content within the tensile zone. Under triaxial compression, confinement dominates: as confining pressure rises from 0 to 30 MPa, compressive strength increases by approximately 32.6%, 52.6%, and 71.3%. Due to crack-suppression by confinement overlapping with fiber bridging, the contribution of fibers to strength gains decreases with increasing confinement, and the competing and complementary interaction between coarse aggregate and steel fibers correspondingly weakens. These findings clarify the coupled effects of coarse aggregate and fibers in UHPC-CA, guide mix-design optimization for improved mechanical performance, and support broader practical adoption.

## 1. Introduction

Ultra-high performance concrete (UHPC), first conceptualized by De Larrard in 1994 based on the particle close-packing theory, has been widely studied for its high strength and durability [1,2,3,4,5,6,7]. These characteristics primarily arise from its high density [8], low porosity, and the use of high-quality raw materials with optimized gradation and minimal impurities [9,10,11]. To ensure matrix homogeneity, conventional UHPC mix designs typically exclude coarse aggregates (CA) [12]. However, this omission substantially increases cement consumption, leading to higher hydration heat, autogenous shrinkage [13], reduced stiffness, elevated costs, and environmental burdens-outcomes inconsistent with current low-carbon development goals [14,15]. Incorporating CA has therefore emerged as a promising strategy to mitigate these drawback [16].

Evidence indicates that CA can reduce binder demand and production cost, restrain autogenous shrinkage, and improve compressive strength and elastic modulus [17]. At the same time, the use of CA introduces design trade-offs whose mechanisms are not fully resolved. In normal-strength (NC), high-strength (HSC), and self-compacting concrete (SCC), CA content, type, size, shape, grading, and quality are known to strongly affect mechanical behaviors [18,19,20]. In UHPC, studies of CA dosage [21,22,23,24], mineralogy [23], and size [9,25] generally report gains in compressive behavior, whereas findings for flexural performance are mixed: several reports show degradation with CA presence or higher contents [26,27,28], while others identify an approximate optimal window that maintains or even improves flexural response [29]. Most of the available literature [20,24,30] suggests that incorporating coarse aggregates with particle sizes of 5–10 mm at dosages of 400–600 kg/m^3^ improves the compressive strength and elastic modulus of UHPC. In terms of flexural behavior, a content range of 200–400 kg/m^3^ generally results in no substantial deterioration in performance. This phenomenon may be attributed, on the one hand, to the type of loading, and on the other hand, to the alterations in fiber dispersion induced by the incorporation of coarse aggregates.

Consistent with this interpretation, other studies show that optimized CA content and grading can benefit UHPC, whereas excessive or poorly graded CA tends to degrade performance-often via impaired fiber dispersion [23,31]. UHPC’s flexural and tensile capacity, including post-cracking ductility, relies on steel fibers that bridge cracks and enhance toughness [32,33,34]; however, CA incorporation can disturb fiber dispersion and orientation within the matrix [33]. Li et al. [35] found that increasing CA content and particle size decreased flexural strength and ductility, and attributed this degradation to disrupted fiber distribution. Similarly, Wang et al. [29] reported that, at higher CA contents, non-uniform fiber dispersion weakened crack-bridging, leading to significant reductions in peak load and deflection. Zhang et al. [24] showed that incorporating CA lowers the fiber-dispersion and orientation coefficients, thereby impairing the tensile and flexural performance of UHPC. They further proposed that, to promote uniform dispersion and avoid agglomeration, carefully graded CA should be combined with fibers whose length-to-diameter ratios are appropriately matched.

Collectively, these observations underscore fiber dispersion as a key mediator of CA effects on UHPC performance across loading regimes. Yet two limitations emerge from the literature. First, most studies consider CA addition or fiber reinforcement in isolation, offering limited insight into their coupled interaction. Second, quantitative links between CA-induced fiber spatial patterns and macroscopic response-load-carrying capacity, deformation behavior, and energy absorption-remain sparse, particularly across distinct loading regimes. These gaps impede the development of UHPC-CA with stable performance and constrain its prospects for engineering application. To address these limitations, this study systematically examines the effects of coarse aggregate incorporation on the mechanical performance of UHPC under multiple loading regimes, including four-point bending, uniaxial compression, and conventional triaxial compression. The spatial distribution of steel fibers within the matrix is quantitatively characterized using image-based analysis. Particular attention is paid to how CA-induced changes in fiber dispersion and orientation relate to load-bearing capacity, deformation response, and energy absorption. On this basis, the mechanisms governing fiber–aggregate interactions and their contributions to mechanical performance across loading conditions are elucidated, providing new insight into the coupled effects of CA and fibers in UHPC-CA systems and a defensible basis for rational UHPC-CA mix design.

## 2. Materials and Methods

### 2.1. Raw Materials

**Figure 1:**
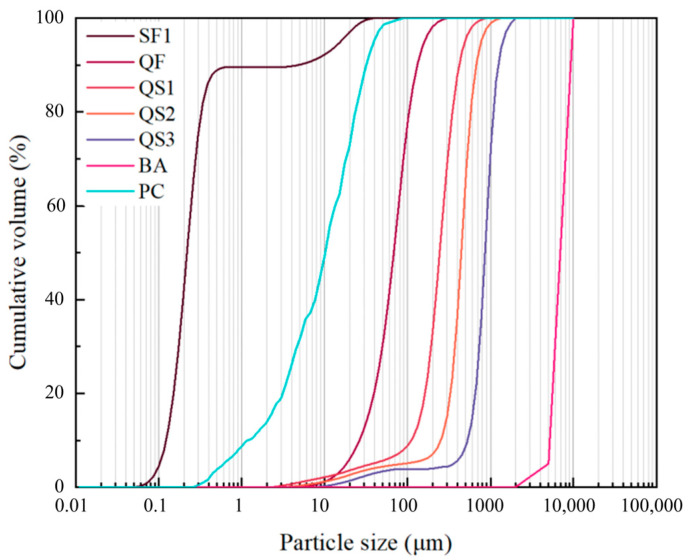
Particle size distribution of raw materials [33].

The raw materials used in this study are Portland cement P·O 52.5 (PC, Chongqing, China), silica fume (SF1, Chongqing, China), quartz flour (QF, Hebei, China), three kinds of quartz sands with different particle sizes (QS1, QS2, and QS3, Hebei, China), basalt aggregate 5–10 mm (BA, Chongqing, China), tap water (W), and PCE-type superplasticizer (SP, Jiangsu, China). The particle size distributions of PC, SF1, QS1, QS2, and QS3 are measured by laser diffraction analyses (Malvern Mastersizer 2000^®^, Malvern, UK), whereas the particle size distribution of BA is measured by sieving. Figure 1 shows the particle size distribution curves of raw materials [33]. The chemical composition of the powders, determined by X-ray fluorescence (XRF), is listed in Table 1. Straight steel fibers (SF, Henan, China) with dimensions of 12 mm in length and 0.2 mm in diameter were used.

### 2.2. Mix Proportions

All the mixtures were designed using the modified Andreasen and Andersen model (MAA). The recipes of UHPCs used in this study are shown in Table 2.

(1) $$P(D)=(D^{q}-D_{\min}^{q})/(D_{\max}^{q}-D_{\min}^{q})$$

(2) $$RSS={\sum_{i=1}^{n}\left[P_{mix}(D_{i}^{\left(i+1\right)})-P_{\mathrm{target}}(D_{i}^{\left(i+1\right)})\right]}^{2}\to min$$

where *P*(*D*) is a fraction of the total solids being smaller than size *D*, *D* is the particle size, *D_max_* is the maximum particle size, *D_min_* is the minimum particle size, *P_mix_* is the composed mix, and *P_target_* is the target grading calculated from Equation (1), *q* is the distribution modulus, and its value depends on the type of concrete being designed. According to the recommendation of Li [15], the value of *q* was selected as 0.19 in this study.

### 2.3. Specimen Preparation and Curing

A 60 L forced single-axis mixer was employed to produce the blend. The procedure began with dry materials being mixed for around 2 min. Next, water combined with a superplasticizer was slowly incorporated, followed by mixing for another 8 min. Lastly, fibers were added into the mixture and blended for an extra 3 min.

UHPC mixtures were poured into molds measuring 50 mm × 100 mm cylindrical for compressive s test, 100 × 100 × 400 mm^3^ for four points bending test, and dog-bone-shaped for single fiber pull-out test. After casting, the samples were immediately covered with plastic wrap and cured at room temperature for 2 days before demolding. Subsequently, they underwent steam curing at 90 °C for an additional 3 days and were tested at 5 days of age.

### 2.4. Testing Methods

#### 2.4.1. Four-Point Bending Test

The four-point bending test was conducted in accordance with the ASTM C1609 [36] standard. Prismatic specimens measuring 100 × 100 × 400 mm^3^ were prepared, with a net span of 300 mm. To ensure accurate deflection measurement at the mid-span, two linear variable displacement transducers (LVDTs) were installed on opposite sides of each specimen. The tests were carried out under displacement control, using a loading rate of 0.15 mm/min.

**Figure 2:**
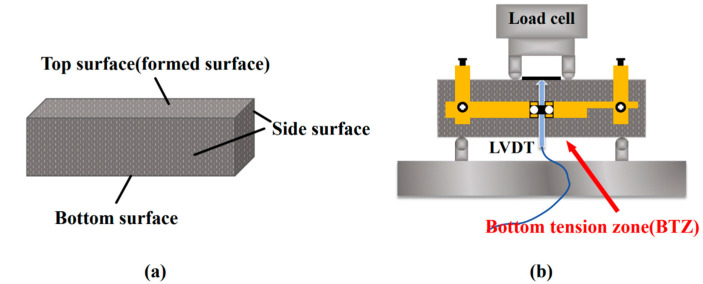
Flexural test setup [33]. (**a**) Schematic diagram of the specimens, (**b**) Loading schematic.

To evaluate the direct influence of steel-fiber distribution on the mechanical performance of UHPC-CA, flexural tests were performed with different faces oriented as the bottom tensile zone, thereby exploiting gravity to modulate the spatial distribution of the fibers. Three distinct surface regions of the specimens-namely the top, bottom, and side-were designated as the bottom tension zone (BTZ). Prior to testing, the specimen surfaces were ground flat to eliminate irregularities and reduce the risk of stress concentration. The complete testing setup is illustrated in Figure 2.

#### 2.4.2. Compressive Strength Test

**Figure 3:**
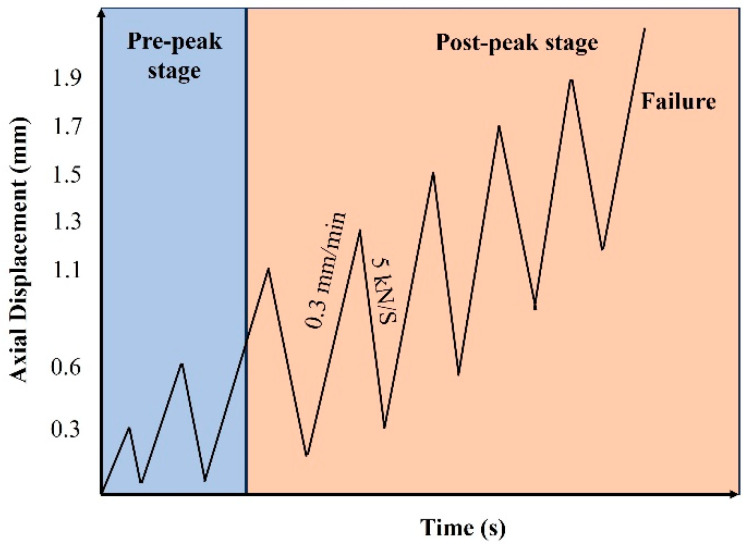
The loading procedures.

All tests of UHPC specimens were performed using MTS-815 electro-hydraulic servo rock testing system (Boston, MA, USA). The confining pressure loading rate is 5 MPa/min. The loading procedures is shown in Figure 3. Axial loading is performed under displacement control at a rate of 0.3 mm/min, while unloading is conducted under force control at a rate of 5 kN/s. The loading rate is relatively low, indicating quasi-static loading conditions.

#### 2.4.3. Fiber Dispersion Test

**Figure 4:**
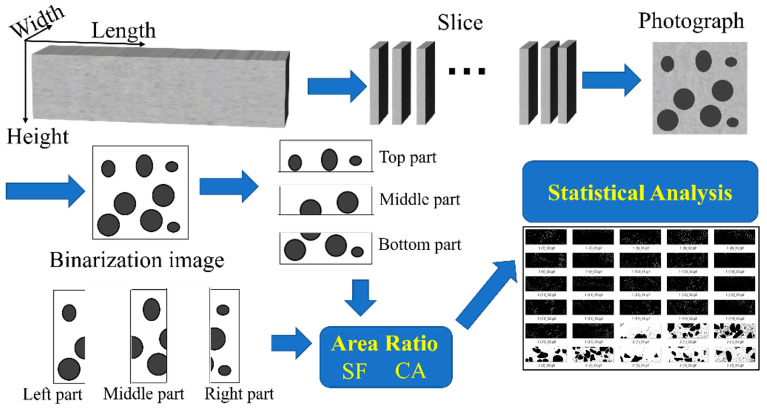
Schematic diagram of coarse aggregate dispersion analysis [33].

Image analysis method [37] was employed to investigate the fiber dispersion, elaborated in Figure 4 [33]. The fiber dispersion evaluation is typically expressed in terms of the fiber distribution coefficient α (Equation (3)) and orientation coefficient θ (Equation (4)).

(3) $$\alpha =exp\left\lfloor -\frac{1}{x_{0}}\sqrt{\frac{\sum {\left(x_{i}-x_{0}\right)}^{2}}{n}}\right\rfloor$$

(4) θ=arccos(D/L)

where *n* is the number of units, here 16; *x_i_* represents the number of fibers in the *i*-th unit, *x*_0_ is the average number of fibers in each unit, *D* is the fiber diameter, and *L* is the long axis of inclined fibers.

## 3. Results

### 3.1. Flexural Properties

#### 3.1.1. Load-Deflection and Key Parameters

**Figure 5:**
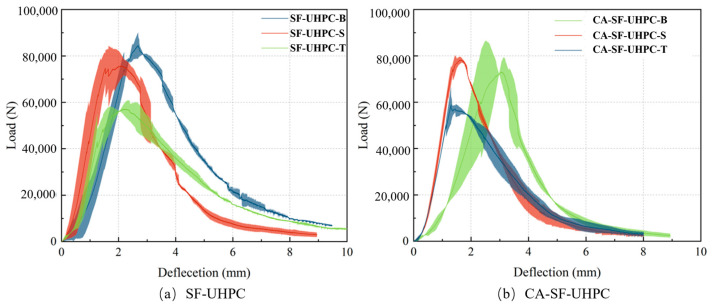
Load-deflection curve of UHPC.

Figure 5 presents the load-deflection responses of UHPC specimens subjected to four-point bending, where different surfaces were positioned as the tension zone. The suffixes -B, -S, and -T denote specimens tested with the bottom, side, and top faces, respectively, positioned in the tensile zone. As shown in Figure 5a, for UHPC reinforced solely with steel fibers, the load-deflection curves exhibit a consistent trend. Regardless of which surface was placed under tension, the specimens demonstrated a ductile failure mode, which can be attributed to the bridging and crack-arresting effects of the steel fibers [38].

Figure 5b illustrates the behavior of UHPC incorporating both coarse aggregate and steel fibers. Although the inclusion of coarse aggregate modifies the internal matrix structure, the overall failure mode remains ductile. This observation suggests that steel fibers are the primary contributor to ductility and crack resistance, whereas the influence of coarse aggregate on failure characteristics is relatively limited. By comparing the three curves corresponding to the same mixture, it can be observed that for both the SF-UHPC and CA-SF-UHPC groups, the curve is consistently lower when the top surface (the casting surface) is placed in the tensile zone. In contrast, the curves for the other two orientations, where the side and bottom surfaces are in tension, are relatively close to each other.

**Figure 6:**
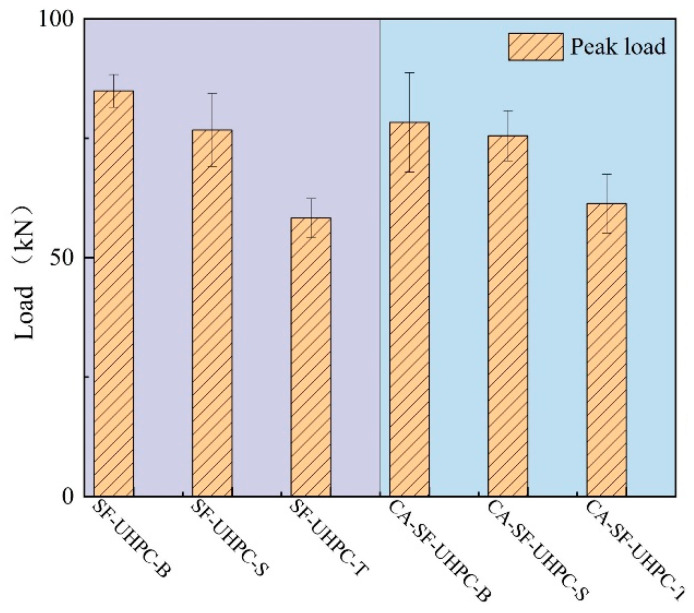
Peak load of UHPCs.

Based on the load-deflection curves, the peak load was extracted and are presented in Figure 6. For both SF-UHPC and CA-SF-UHPC specimens, the highest peak load occurs when the bottom surface is placed in tension, followed by the side surface, and finally the top (casting) surface. When comparing the two mixtures, the addition of coarse aggregate results in a pronounced reduction in peak load for specimens tested with the bottom surface in tension and a slight reduction for those with the side surface in tension. In contrast, the peak load increases when the top surface is in tension. Moreover, the variation in peak load among different loading orientations becomes less significant after incorporating coarse aggregate, suggesting a more uniform structural response.

Our previous work [33] reported the variations in UHPC toughness when different faces served as the bottom tensile zone. The toughness index Ts150 primarily reflects the area under the load-deflection curve up to the peak load, while Ts50 corresponds to the area up to the inflection point. For both groups, Ts150 and Ts50 exhibit a consistent trend: the highest values are obtained when the side surface is under tension, followed by the bottom surface, with the lowest values observed for the top surface. The incorporation of coarse aggregates leads to a significant reduction in toughness indices. Moreover, when different surfaces are subjected to tension, the CA-SF-UHPC group shows smaller variations in toughness compared to the SF-UHPC group. This behavior may be attributed to the modified fiber orientation and aggregate interlocking effect caused by the presence of coarse aggregate, which alters crack propagation paths and energy dissipation mechanisms. These findings suggest that steel fibers play a critical role in governing the failure behavior of UHPC, whereas the incorporation of coarse aggregates is the dominant factor contributing to the reduction in toughness.

#### 3.1.2. Distribution of Fiber

Table 3 summarizes the fiber distribution coefficients and orientation angles of steel fibers in both SF-UHPC and CA-SF-UHPC groups. According to the calculation method of the distribution coefficient, it is evident that the coefficient remains constant regardless of which surface is subjected to tension. The orientation angle represents the angle between the fiber and the loading direction. Since rotating the specimen does not alter the angle between the fiber plane and the vertical direction, the orientation angle remains unchanged. The fiber distribution results indicate that the incorporation of coarse aggregates leads to a slight increase in both the distribution coefficient and the orientation angle. This finding, particularly the increased distribution coefficient after coarse aggregate inclusion, deviates from conclusions reported in previous studies. A possible explanation lies in the sedimentation of steel fibers.

**Figure 7:**
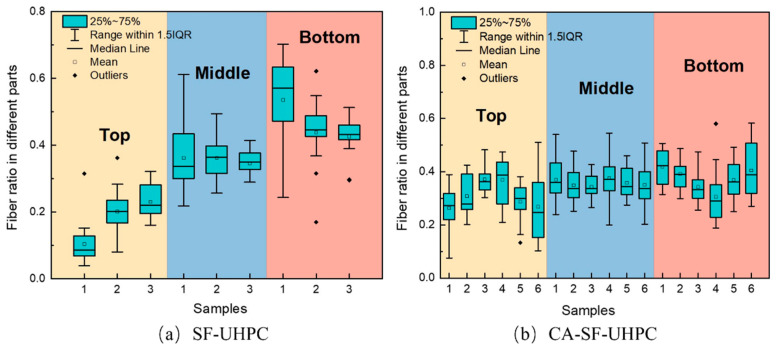
Fiber distribution of UHPCs [33].

To investigate this hypothesis, Figure 7 shows the distribution of fibers in the top, middle, and bottom sections of the specimens. As seen in Figure 7a, the proportion of fibers in the top region is notably lower than that in the bottom, suggesting that fiber settlement occurred during casting. Similar trends have been observed in the literature [39], often attributed to the rheological properties of the fresh paste and the mixing or casting procedures. As shown in Figure 7b, the incorporation of 10 mm coarse aggregates at a volume fraction of 20% alleviated fiber settlement, leading to a more uniform fiber distribution throughout the specimen height. Consequently, in this study, the addition of coarse aggregates unexpectedly led to an improvement in the fiber dispersion coefficient in UHPC.

### 3.2. Compressive Properties

#### 3.2.1. Stress–Strain

**Figure 8:**
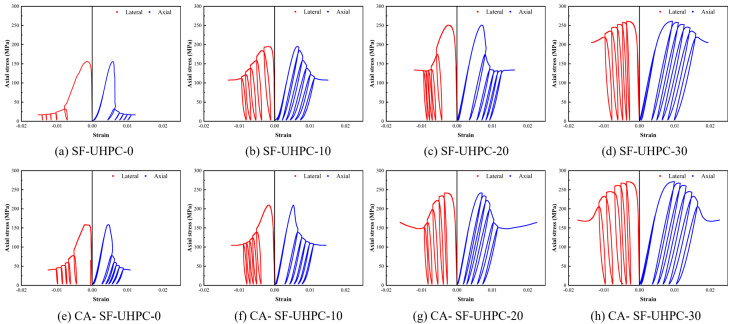
Stress–strain curve of UHPCs.

To investigate the influence of coarse aggregates on UHPC under different loading conditions, both uniaxial compression and conventional triaxial compression tests were conducted in this study. The triaxial tests were performed under three levels of confining pressure, 10 MPa, 20 MPa, and 30 MPa. Figure 8 presents the stress–strain curves of SF-UHPC and CA-SF-UHPC specimens under different confining pressures. As shown in the figure, with increasing confining pressure, the stress–strain curves become both higher and broader. This behavior can be attributed to the Poisson effect. Under axial loading, tensile stresses develop in the lateral direction, and the application of confining pressure helps to counteract these tensile stresses. As a result, both the compressive strength and the deformation capacity of the specimens increase. By comparing the upper and lower rows of Figure 8, it is evident that the addition of coarse aggregates has only a minor effect on the stress–strain response, suggesting that their influence is limited under the tested conditions.

**Figure 9:**
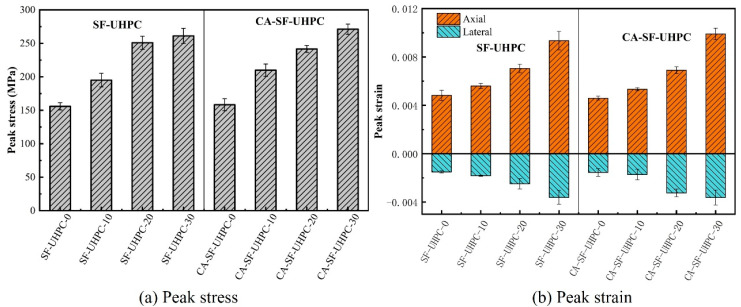
Peak stress and peak strain of UHPCs.

The peak stress and the corresponding axial and lateral strains extracted from the stress–strain curves are shown in Figure 9a,b, respectively. As illustrated in Figure 9a, the peak stress increases with the rise in confining pressure. For the SF-UHPC group, the peak stress increases by 34.6%, 60.9%, and 67.5% at confining pressures of 10 MPa, 20 MPa, and 30 MPa, respectively. For the CA-SF-UHPC group, the corresponding increases are 32.6%, 52.6%, and 71.3%. It is worth noting that the increase in peak stress is not linear; the rate of increase diminishes as the confining pressure rises, suggesting the existence of a threshold beyond which the effect of confining pressure on UHPC strength becomes less significant. This trend is consistent with previous findings in the studies [40,41,42], which attribute the phenomenon to the combined roles of fibers and confining pressure in resisting tensile stresses. As the confining pressure increases, it reduces the contribution of the fiber bridging effect, indicating that the enhancement effect of confinement becomes less significant at higher levels.

Figure 9b shows the variation in peak axial and lateral strains with increasing confining pressure. Both axial and lateral strains increase as the confining pressure increases. For the SF-UHPC group, the axial peak strain increases by 16.1%, 45.9%, and 93.6%, while for the CA-SF-UHPC group, the corresponding increases are 16.1%, 50.3%, and 115.7%. In terms of lateral peak strain, the SF-UHPC group exhibits increase of 20.5%, 64.9%, and 139.1%, and the CA-SF-UHPC group shows increases of 13.9%, 60.4%, and 132.9%, respectively. Unlike the trend observed for peak stress, the peak strain exhibits an accelerating growth pattern with increasing confining pressure. This suggests that, while the strength gain becomes less pronounced with increasing confinement, the deformation capacity continues to show substantial improvement.

#### 3.2.2. Distribution of Fiber

Table 4 presents the distribution coefficients and orientation angles of steel fibers in UHPC. It can be observed that for specimens with the same mix design, variations in fiber distribution coefficient and orientation angle exist due to the inherent randomness of fiber dispersion. Moreover, the data indicate that the inclusion of coarse aggregates in UHPC generally results in a decrease in distribution coefficients and an increase in orientation angles.

Interestingly, this trend contrasts with the findings observed in the bending test, where the addition of coarse aggregates was found to improve fiber dispersion. This discrepancy is primarily attributed to the sampling method. Specimens for the triaxial compression test were extracted via core drilling, representing only a partial volume of the original specimen. As a result, the suppression of fiber settlement by coarse aggregates is not fully captured in these samples. However, the presence of coarse aggregates limits the spatial freedom for fibers to orient in other directions, which may lead to a reduction in the overall distribution coefficient.

## 4. Discussion

### 4.1. Effect of Fiber Distribution on Four-Point Bending Response of UHPC

**Figure 10:**
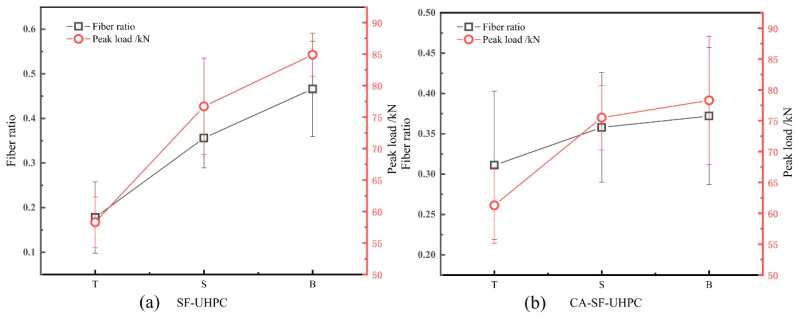
Correlation between fiber volume fraction in the tension zone and flexural strength.

**Figure 11:**
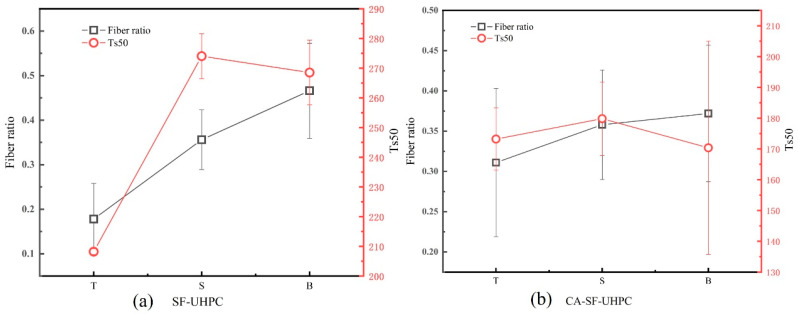
Correlation between fiber volume fraction in the tension zone and flexural toughness.

Based on the results presented in Section 3.1, it was found that while the overall distribution coefficient and orientation angle of steel fibers in UHPC remained relatively unchanged when different surfaces were chosen as tensile zone, the resulting flexural strength and toughness exhibited significant variation. To further investigate this phenomenon, the proportions of steel fibers in the upper, middle, and lower regions of the specimens, along with the corresponding peak load values, are illustrated in Figure 10. Additionally, Figure 11 presents the fiber content and toughness index Ts50.

As shown in Figure 10, both SF-UHPC and CA-SF-UHPC display a consistent trend between the proportion of fibers in the tensile zone and the corresponding peak load under four-point bending. Specifically, an increase in the proportion of steel fibers located within the tensile zone leads to a corresponding increase in the peak load. This indicates that the flexural strength of UHPC is strongly influenced by the amount of steel fibers present in the tension zone-higher fiber content in this region enhances the UHPC’s resistance to bending loads. This observation is consistent with prior reports that flexural/tensile response in UHPFRC is governed by crack-bridging fibers and their effective engagement in the tensile zone [4,43,44]. These findings highlight the critical role of fiber localization within the tensile region in optimizing the flexural performance of UHPC, even when the overall fiber distribution appears similar across specimens.

As shown in Figure 11, the trend of the toughness index Ts50 does not fully align with the variation in the steel fiber content within the tensile zone. When the top surface serves as the tensile region, both the fiber proportion and Ts50 reach their lowest values. As the tensile region shifts to the side surface, both parameters increase. However, when the bottom surface is subjected to tension, although the fiber content continues to increase, Ts50 begins to decline. This indicates that the toughness index is not solely governed by the quantity of fibers in the tensile zone, but also influenced by factors such as fiber orientation, dispersion uniformity, and the effectiveness of stress transfer during crack propagation. The sensitivity of toughness to orientation and pull-out mechanics is well documented, especially for inclined fibers that shorten the embedded length and reduce pull-out resistance [45,46,47].

**Figure 12:**
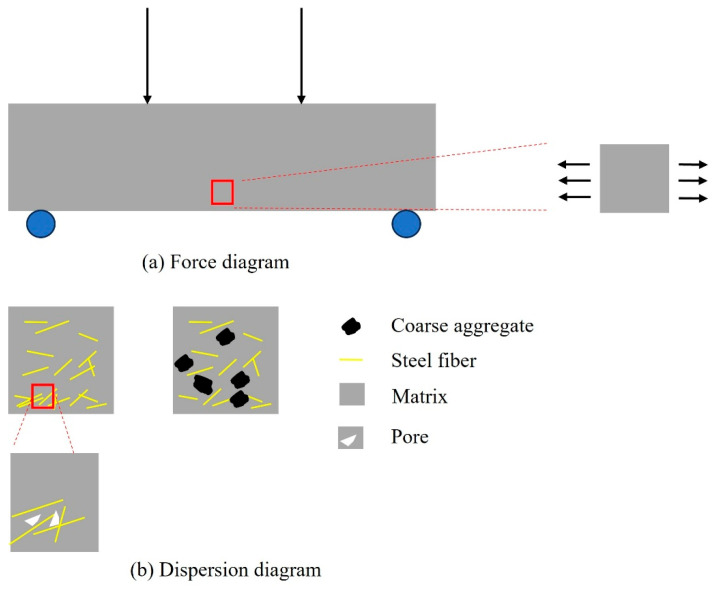
Schematic of loading and dispersion.

Figure 12 illustrates both the loading condition and the schematic of fiber dispersion. Under flexural loading, the bottom region of the specimen is subjected to tensile stress. In UHPC, a large quantity of steel fibers can effectively resist this tensile force. As a result, when the bottom surface is under tension, the content of steel fibers contributing to tensile resistance is at its highest, leading to the greatest flexural strength. Conversely, the top region contains the fewest steel fibers; thus, when it is under tension, the corresponding flexural strength is the lowest.

With the incorporation of coarse aggregates, two major effects are observed. First, the aggregates disrupt the continuity of fiber distribution. Second, they occupy the available space for fiber dispersion, thereby reducing the number of fibers that effectively contribute to tensile resistance. Additionally, the presence of coarse aggregates increases the average orientation angle of the fibers, weakening their bridging capacity across cracks. However, the addition of coarse aggregates also suppresses the downward migration (settling) of steel fibers during casting, resulting in a more uniform distribution and an increased fiber content near the top surface. Consequently, when the top surface is in tension, the CA-SF-UHPC exhibits higher flexural strength than the SF-UHPC without coarse aggregates. Similar CA-induced redistribution-raising orientation angles and disrupting continuity-has been shown to diminish crack-bridging efficiency and flexural ductility in UHPC; imaging/CT-based assessments also captured settlement and near-surface enrichment [39,43].

On the other hand, the decrease in fiber continuity and the increase in orientation angle led to lower energy consumption during the fiber pull-out process, which accounts for the reduced toughness. Moreover, excessive fiber clustering can introduce initial defects, such as poor bonding between adjacent fibers and the matrix. This lack of effective anchorage reduces bond-friction resistance during pull-out, thereby lowering energy absorption [45,48,49,50]. Therefore, despite the higher fiber content near the bottom, the toughness of UHPC may decline when the bottom surface serves as the tensile region.

### 4.2. The Role of Confining Pressure and Fibers in the Compressive Behavior of UHPC

In the compression test, the influence of confining pressure is inherently present and has a significant effect on the overall mechanical response. As a result, it is not feasible to isolate and directly evaluate the effects of fiber dispersion coefficient and orientation angle under compressive loading. Therefore, the analysis must rely on the fiber mechanisms and stress conditions identified from the flexural test results.

**Figure 13:**
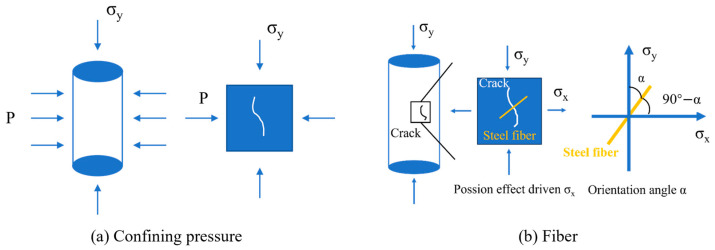
Schematic of confining pressure and fiber action mechanisms.

Figure 13a,b illustrates the roles of confining pressure and steel fibers in the compressive behavior of UHPC. Under axial loading, due to the Poisson effect, lateral tensile stresses are generated within the material, which may lead to the initiation and propagation of microcracks. As shown in Figure 13a, the application of confining pressure counteracts these lateral tensile stresses, suppresses crack formation, and promotes the closure of existing cracks, thereby enhancing the compressive strength. Meanwhile, the role of steel fibers in resisting compressive damage is primarily achieved through their ability to bridge cracks via bond and frictional resistance during fiber pull-out. As depicted in Figure 13b, when the fiber orientation angle increases, the effective embedded length decreases, reducing the fiber’s pull-out resistance. Moreover, when the angle exceeds approximately 60°, the pull-out process tends to cause significant damage to the surrounding matrix, which in turn diminishes the fiber bridging effectiveness [51].

When confining pressure is applied during compression, its crack-suppressing function overlaps with that of the steel fibers. This redundancy weakens the relative contribution of the fibers, especially in terms of crack bridging. This interaction explains the trend observed in Section 3.2.1: as the level of confining pressure increases, the rate of improvement in compressive strength gradually decreases. Similar overlaps between confinement-driven crack suppression and fiber bridging-leading to diminishing fiber contribution at higher pressures-have been discussed in recent triaxial studies [52,53].

## 5. Conclusions

Based on the experimental results and analysis, the following conclusions were obtained:

1. Tension-zone fibers govern flexural response. Flexural capacity and post-cracking toughness are governed predominantly by the local steel-fiber fraction within the tensile zone.
2. Coarse aggregate has a relatively small influence on both flexural and compressive strength but a pronounced influence on toughness. Although its addition reduces flexural toughness, it also narrows the differences in measured toughness among specimens tested with different faces serving as the tensile zone.
3. CA addition mitigates fiber settlement. Introducing CA (10 mm, 20 vol%) attenuates fiber settlement and modestly improves vertical uniformity: the distribution coefficient increases from 0.651 to 0.673, while the orientation angle rises from 48.77° to 48.90°.
4. Triaxial compression behavior is confinement-dominated. Due to the overlapping crack suppression mechanisms, the contribution of steel fibers to strength enhancement is gradually diminished as confinement increases.
5. Coarse aggregate and steel fibers have both complementary and competing effects. Coarse aggregate limits fiber settlement but disrupts fiber continuity. When confinement is present, the confining pressure diminishes their complementary and competing interaction.

These findings provide a mechanistic, data-supported basis for the design of UHPC-CA. They inform mix design and placement procedures that suppress fiber settlement while preserving crack-bridging efficiency. In engineering practice, the flexural behavior and the compressive response offer clear design guidance for performance tailoring and quality control of structural members.

## Figures and Tables

**Table 1:** Chemical composition of the Portland cement and silica fume [33] (wt%).

	SiO_2_	Al_2_O_3_	CaO	Fe_2_O_3_	SO_3_	MgO	Na_2_O	K_2_O	LOI
Cement	19.41	4.32	65.29	3.06	2.89	2.47	0.068	0.71	2.3
Silica fume	91.87	0.21	0.19	0.12	1.68	3.05	0.073	0.18	1.6
Basalt aggregate	49.11	15.82	10.57	11.20	-	7.13	2.27	0.70	-

**Table 2:** Recipes of UHPCs [33] (kg/m^3^).

Specimen Name	C	SF1	QF	QS	BA	W	SP	SF	W/C
SF-UHPC	685	60	220	1165	0	150	26.1	195	0.2
CA-SF-UHPC	695.7	31.6	79	977	600	145.5	25.5	195	0.2

**Table 3:** Fiber distribution coefficient and orientation angle of UHPCs.

Group	Distribution Coefficient	Orientation Angle (°)
SF-UHPC	0.651	48.77
CA-SF-UHPC	0.673	48.90

**Table 4:** Distribution coefficient and orientation angle of UHPCs.

Group	Distribution Coefficient	Orientation Angle (°)
SF-UHPC-0	0.644	48.55
SF-UHPC-10	0.664	44.52
SF-UHPC-20	0.744	43.03
SF-UHPC-30	0.627	44.77
CA-SF-UHPC-0	0.599	43.33
CA-SF-UHPC-10	0.502	48.01
CA-SF-UHPC-20	0.491	48.16
CA-SF-UHPC-30	0.665	48.12

## Data Availability

The original contributions presented in this study are included in the article. Further inquiries can be directed to the corresponding authors.
